# Editorial: Cancer Metabolism: Molecular Targeting and Implications for Therapy

**DOI:** 10.3389/fonc.2017.00232

**Published:** 2017-09-26

**Authors:** Shanmugasundaram Ganapathy-Kanniappan

**Affiliations:** ^1^Division of Interventional Radiology, Department of Radiology and Radiological Sciences, Johns Hopkins University School of Medicine, Baltimore, MD, United States

**Keywords:** cancer metabolism, metabolic reprogramming, glycolysis, immunotherapy, tumor microenvironment

**Editorial on the Research Topic**

**Cancer Metabolism: Molecular Targeting and Implications for Therapy**

Metabolic reprogramming, *sensu stricto*, refers to rewiring of metabolic circuitry, which in turn involves alteration of multiple energy producing pathways (1). However, until the recent couple of decades, only the glucose metabolism has been the subject of extensive investigation, partly due to the early observation of the distinctive biochemical phenotype, i.e., rapid rate of glucose utilization, the phenomenon that is currently exploited in the clinical diagnosis of cancer by PET imaging (2). Nevertheless, it is increasingly evident that besides glucose metabolism, other metabolic pathways like glutamine metabolism and lipid/fatty acid metabolism among others also play critical roles in cancer growth. Thus, a repertoire of alterations occur during the metabolic reprogramming of cancer. Hence, the “deregulation of bioenergetics” has recently been recognized as one of the hallmarks of cancer (3). The objective of this Frontiers research topic is to advance our current understanding of cancer metabolism in its entirety to achieve the development of a viable and effective strategy for cancer therapy. The research topic begins with four reviews, three mini-reviews followed by three original research articles.

Weber has elegantly presented the metabolic phenotype of cancer during various stages of the disease progression. The review outlines a global perspective on cancer metabolism encompassing both intrinsic and extrinsic factors and their impact on the metabolic phenotype. More importantly, Weber has deciphered the potential differences in metabolic phenotype that could distinguish various stages of tumors such as “primary tumors (Warburg effect), metastasizing cancer cells (peroxide-driven ATP production), tumor cells under stromal influence (lactate uptake, inflammation), and late-stage cancers (hypoxia).” Finally, the review emphasizes on the prospects of therapeutic targeting of cancer metabolism and the necessity to consider a combinatorial approach to overcome any potential challenges.

Although metabolic alteration is common and frequent among cancers, variations do exist in the type of metabolic change between benign and malignant neoplasms. By using prostate cancer as a prototype, Siddiqui and colleagues (Eidelman et al.) present a detailed overview of the metabolic characteristics of prostate cancer. In addition to the distinguishing feature of citric acid metabolism between the benign and malignant prostate tumors, the review focuses on other energy-producing pathways as well. Noteworthy, the diagnostic potential and implications of versatile techniques involving metabolomics have also been discussed. In the words of Siddiqui and colleagues, “there is still a great deal of research to be done, as many of the mechanisms of cellular cancer metabolisms are not well understood,” and the review provides a summary of current knowledge on the cancer metabolism of prostate cancer.

It is well known that oncogenic events regulate cancer metabolism either directly or indirectly, through signaling mechanisms. In the review, Nickerson and colleagues (Nickerson et al.) have delineated the functional link between metabolic reprogramming and its epigenetic regulation in cancer. With a focus on the chromatin remodeling enzyme, Brg1, the review describes in detail the molecular intricacies involved in metabolic reprogramming, especially the lipid metabolism. The biochemical and functional significance of Brg1-dependent transcriptional regulation of key lipogenic enzymes in breast cancer has been discussed in detail with reference to reprogramming of cancer-related fatty acid metabolism. Notably, the review also emphasizes on one of the emerging areas of research, the “metaboloepigenetics.”

Multiple lines of evidence demonstrate that the immediate vicinity of tumor, commonly referred as the tumor microenvironment (TME) impacts the metabolic phenotype of cancer. In an extensive review, Dwarakanath and colleagues (Gupta et al.) exquisitely present the functional association between TME and cancer metabolism. Besides cancer cells, the review also discusses the mechanistic insights on the role of TME and its components on cancer-associated immune cells, adipocytes, endothelial cells, and fibroblasts. Dwarakanath and colleagues appropriately state that advances in the complete understanding of metabolic reprogramming and TME may provide an opportunity to develop effective anticancer therapies.

Next, the aerobic glycolysis also known as tumor glycolysis is a prominent metabolic event in majority of solid tumors at least in some stages of the disease. The review entitled, “Taming Tumor Glycolysis and Potential Implications for Immunotherapy” (Ganapathy-Kanniappan) discusses the contributions of tumor glycolysis in escaping immune surveillance and the therapeutic opportunities of its deregulation. Recent data demonstrate that cellular and/or metabolic stress augments the sensitivity of cancer cells to natural killer (NK) cell-mediated cytotoxicity (4, 5). This review emphasizes on the hitherto unexplored immunotherapeutic opportunities of targeting tumor glycolysis. While the focus of the review is on taming tumor glycolysis and its sensitivity to NK cells, in principle, the strategy can be expanded to include any of the major energy-producing pathways of cancer cells. Noteworthy, the objective of disruption of energy-producing pathways is to induce stress that is necessary to upregulate specific stress-inducible ligands for further recognition by NK cells. Thus, the immunotherapeutic potential of targeting cancer metabolism is an emerging area of research that warrants further investigation.

Among several metabolic enzymes, aldo-keto reductases have been known to play an indispensable role in intermediary metabolism, macromolecular biosynthesis, and the detoxification of free radicals. Recent research has shed light on the association between aldo-keto reductases and cancer growth and therapeutic resistance. In the comprehensive review, Yang and colleagues (Zeng et al.) discuss the molecular and biochemical correlations of aldo-keto reductases in breast cancer and prostate cancer. More importantly, the review deliberates on the catalytic function-dependent and function-independent roles of one of the isoforms of aldo-keto reductase as well. The review focuses on the opportunities and therapeutic potential of targeting aldo-keto reductases with small molecule inhibitors and emphasizes on the need for further research.

Arachidonic acid metabolism has only recently been implicated in prostate cancer, and the review by Abou-Kheir and colleagues (Bilani et al.) discusses the effect of disruption of arachidonic acid metabolism by COX inhibition in prostate cancer. By using the example of NSAID, aspirin, the authors discuss the mechanism underlying its anticancer effects in prostate cancer. Although aspirin-dependent antimetastatic effects have been reported, controversies exist in the mechanism that is implicated for such anticancer effects. With the availability of substantial epidemiological data, the authors underscore *al beit* with caution the need for further investigation of aspirin-dependent biochemical or metabolic alterations in prostate cancer to verify any beneficial outcomes of aspirin in prostate cancer.

The next three research articles are related to the anticancer effect of microRNA, i.e., miR-150 (Xu et al.), and the natural product compounds, i.e., AG36 (Mu et al.) and Curcumin (Kumar et al.). These articles advance our understanding of the antiproliferative effects of miR-150, AG36, and Curcumin in leukemia (miR-150) and breast cancer (AG36, Curcumin).

Overall, the collections of reviews and research articles presented under the research topic will enable us to advance our knowledge on cancer metabolism and stimulate critical evaluation of therapeutic targeting of metabolism in cancer.

## Author Contributions

The author confirms being the sole contributor of this work and approved it for publication.

## Conflict of Interest Statement

The author declares that the research was conducted in the absence of any commercial or financial relationships that could be construed as a potential conflict of interest.
